# Chondrosarcoma: A Rare Misfortune in Aging Human Cartilage? The Role of Stem and Progenitor Cells in Proliferation, Malignant Degeneration and Therapeutic Resistance

**DOI:** 10.3390/ijms19010311

**Published:** 2018-01-21

**Authors:** Karen A. Boehme, Sabine B. Schleicher, Frank Traub, Bernd Rolauffs

**Affiliations:** 1 G.E.R.N. Tissue Replacement, Regeneration & Neogenesis, Department of Orthopedics and Trauma Surgery, Medical Center—Albert-Ludwigs-University of Freiburg, Faculty of Medicine, Albert-Ludwigs-University of Freiburg, 79108 Freiburg, Germany; berndrolauffs@googlemail.com; 2 Department of Hematology and Oncology, Eberhard Karls University Tuebingen, Children’s Hospital, 72076 Tuebingen, Germany; sabine.schleicher@med.uni-tuebingen.de; 3 Department of Orthopedic Surgery, Eberhard Karls University Tuebingen, 72076 Tuebingen, Germany; frank.traub@med.uni-tuebingen.de

**Keywords:** chondrosarcoma, articular cartilage, mesenchymal stem and progenitor cell, primary cilia, angiogenesis, metastasis, differentiation, fibroblast growth factor 2, isocitrate dehydrogenase, vascular endothelial growth factor

## Abstract

Unlike other malignant bone tumors including osteosarcomas and Ewing sarcomas with a peak incidence in adolescents and young adults, conventional and dedifferentiated chondrosarcomas mainly affect people in the 4th to 7th decade of life. To date, the cell type of chondrosarcoma origin is not clearly defined. However, it seems that mesenchymal stem and progenitor cells (MSPC) in the bone marrow facing a pro-proliferative as well as predominantly chondrogenic differentiation milieu, as is implicated in early stage osteoarthritis (OA) at that age, are the source of chondrosarcoma genesis. But how can MSPC become malignant? Indeed, only one person in 1,000,000 will develop a chondrosarcoma, whereas the incidence of OA is a thousandfold higher. This means a rare coincidence of factors allowing escape from senescence and apoptosis together with induction of angiogenesis and migration is needed to generate a chondrosarcoma. At early stages, chondrosarcomas are still assumed to be an intermediate type of tumor which rarely metastasizes. Unfortunately, advanced stages show a pronounced resistance both against chemo- and radiation-therapy and frequently metastasize. In this review, we elucidate signaling pathways involved in the genesis and therapeutic resistance of chondrosarcomas with a focus on MSPC compared to signaling in articular cartilage (AC).

## 1. Chondrosarcoma Subtypes and Epidemiology

Chondrosarcomas are rare mesenchymal tumors with a cartilage-like appearance. They account for 10–20% of all malignant bone tumors [1]. In Europe, the incidence of chondrosarcoma is 0.1:100,000 [2]. In comparison, the incidence of osteoarthritis (OA) is 100–200:100,000, with a prevalence of symptomatic OA of about 20% in people aged ≥50 years [3,4].

Indeed, the term chondrosarcoma encompasses a group of tumors, which are heterogeneous both morphologically and clinically. About 80–90% of all chondrosarcomas are conventional chondrosarcomas [5]. Conventional chondrosarcomas start to grow intramedullary (central) and often affect the pelvis, femur and humerus, but also ribs and ilium [6]. The highly aggressive dedifferentiated chondrosarcomas, which make up about 10% of all chondrosarcomas [7] most often arise in the long bones, namely the femur and humerus or the pelvis [8]. Dedifferentiated chondrosarcomas consist of chondroid and non-chondroid parts often resembling fibroblastic or osteoblastic tissue indicating two types of mesenchymal differentiation in one tumor [9,10]. Notably, the non-cartilaginous part of these tumors seems to determine local growth and recurrence [11].

Secondary chondrosarcomas may develop from formerly benign central cartilage lesions like enchondromas [12]. In addition, peripheral chondrosarcomas may arise from the cartilaginous cap of osteochondromas, which are benign bone tumors of childhood and adolescence [13,14]. Moreover, there are very rare low-grade entities like clear cell chondrosarcomas, which often involve the epiphysis of the proximal femur and humerus and extend to the articular cartilage (AC) of the acetabulofemoral joint and glenohumeral joint [15,16].

Unlike other bone sarcomas, conventional and dedifferentiated chondrosarcomas have their peak incidence at the ages of 40–70 years [8,17]. In contrast, clear cell chondrosarcomas and peripheral chondrosarcomas more often affect adolescents and young adults [15]. 

Low grade chondrosarcomas rarely metastasize. In the 2013 World Health Organization (WHO) classification system, grade I chondrosarcomas have been renamed as atypical cartilaginous tumors describing their clinical behavior as an intermediate type of tumor [1,18,19]. In contrast, high grade chondrosarcomas, which make up 5–10% of all conventional chondrosarcomas, are very aggressive and frequently metastasize to the lung [5] with a five-year survival rate of 50–60% and a ten-year survival rate of only 30–40% [6]. Yet, also about 20% of low grade tumors locally recur [6]. 

## 2. Mesenchymal Stem and Progenitor Cells in Adult Bone and Cartilage

Mesenchymal stem and progenitor cells (MSPC) exhibiting multipotent differentiation potential are resident in the bone marrow [20]. Although, the cells of origin of chondrosarcoma are still not clearly defined [21,22], conventional and dedifferentiated chondrosarcomas most likely develop from MSPC in the medullary space of bones [23]. 

According to the International Society for Cellular Therapy (ISCT), bone marrow derived mesenchymal stem cells (MSC) must differentiate to osteoblasts, adipocytes and chondrocytes in vitro and should at least express the markers cluster of differentiation 73 (CD73), CD90 and CD105, while markers characteristic for monocytes (CD11b or CD14), B cells (CD19 or CD79a), hematopoietic stem cells (HSC) (CD34), leukocytes (CD45 or human leukocyte antigen—antigen D related (HLA-DR)) are absent [20,24]. In addition, stromal cell surface marker-1 (STRO-1) and CD106 positive MSPC have a high proliferative potential [25]. Indeed, MSPC express a mixture of markers which undergo dynamic changes according to growth factor and cytokine availability during development and disease or artificial plastic adherence in cell culture [26,27]. In addition, MSPC marker expression depends on the species [27].

**Figure 1:**
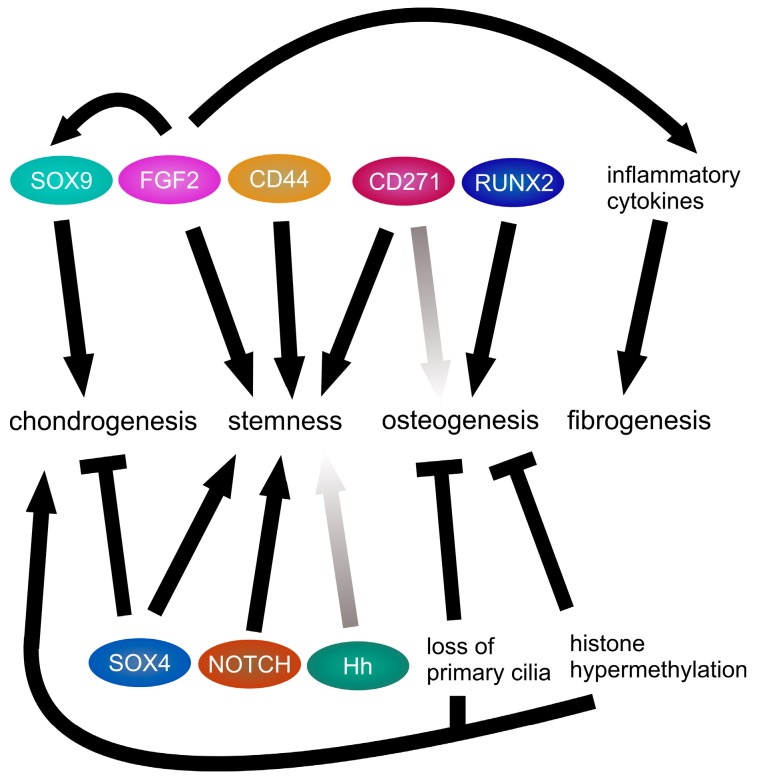
Conflicting differentiation stimuli in chondrosarcoma. In chondrosarcoma cells signaling pathways simultaneously promoting or antagonizing chondrogenesis, stemness, osteogenesis and fibrogenesis are activated preventing differentiation in one or the other direction. Established positive stimuli are depicted as black arrows, established inhibitory stimuli are depicted as black bar-headed lines. Pathways, which are active in chondrosarcoma cells, but whose stimulatory function has not been clearly established in chondrosarcoma are shown as light gray arrows.

Actually, chondrosarcomas express several proteins either known as MSPC markers, chondrogenic markers or markers of other mesenchymal lineage commitment (Figure 1). From primary conventional chondrosarcomas, two cell types with different marker expression signatures have been isolated. One group resembled multipotent MSC (CD49b high/CD10 low/CD221, also known as insulin-like growth factor 1 receptor (IGF1R), high), whereas a second group more likely corresponded to a fibroblastic lineage (CD49b low/CD10 high/CD221 low). This implicates that both chondrosarcoma cell types arose from MSPC, which are the assumed origin of chondrosarcomas [23].

**Figure 2:**
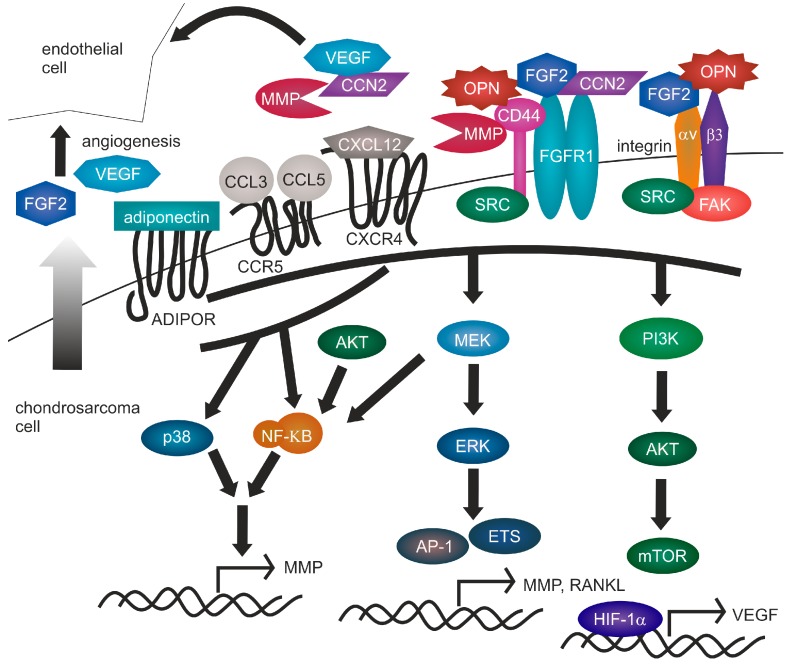
Chondrosarcoma signaling. Several growth factor and cytokine regulated signaling pathways are activated in central chondrosarcomas (black arrows). FGFR1, integrins, ADIPOR, CCR5 and CXCR4 are all capable of MAPK-ERK and PI3K-AKT signaling induction leading to MMP, RANKL and VEGF transactivation. Moreover ADIPOR, CCR5 and CXCR4 activate NF-κB and p38 MAPK signaling. In addition, signaling regulation is obtained by adaptor proteins like CCN2, which binds VEGF, FGF2 and FGFR1 or coreceptors including CD44. Chondrosarcoma cells actively excrete FGF2 and VEGF (gray arrow), which promotes angiogenesis by attracting endothelial cells.

CD44, also known as phagocytic glycoprotein 1 (PGP-1) is a tumor stem cell marker and receptor for hyaluronan, osteopontin (OPN), collagens, and matrix metalloproteinases (MMP) in various tissues (Figure 2). Moreover, CD44 may act as a cofactor for vascular endothelial growth factor (VEGF) and fibroblast growth factor 2 (FGF2) binding. After cleavage, its intracellular domain is involved in transactivation of notch homolog 1 (*NOTCH1)*, receptor activator of NF-κB ligand *(RANKL)* and *MMP-9* expression [28]. CD44 overexpression is increased in chondrosarcomas with progressive grading and correlated with metastatic potential and survival [29]. Interestingly, CD44 expression in human MSPC seems to be acquired in culture since freshly isolated MSPC are generally negative for this marker [30,31]. CD271, a stem cell marker, which may be associated with osteogenic potential of MSPC [32], was expressed by a highly proliferative subpopulation of chondrosarcoma cells [33], indicating that sustained stemness may increase chondrosarcoma proliferation. 

Members of the SRY-related HMG box-containing (SOX) family of transcription factors are master regulators of cell differentiation [34,35]. Human conventional chondrosarcomas of all grades express SOX9 [36], which is the main mediator of chondrogenesis [34]. In addition, SOX5 and SOX6 augment the pro-chondrogenic transcriptional activity of SOX9 [37]. MiR-145, which negatively regulates *SOX9*, was downregulated in human chondrosarcomas, enhancing the relative abundance of SOX9 protein [38]. SOX4 is expressed by human MSPC and downregulated during chondrogenic differentiation [39]. Notably, *SOX4* and runt related transcription factor 2 *(RUNX2)*, which are both implicated in regulation of chondrogenesis and osteogenesis, are targets of miR-30a, which is progressively downregulated with increasing chondrosarcoma grade, leading to a relative overexpression of SOX4 and RUNX2 [40]. Also, miR-129-5p, which targets *SOX4*, was significantly downregulated in human chondrosarcoma tissues, while SOX4 protein was activated [41]. Indeed, this imbalance of SOX9 and SOX4 may contribute to the incomplete chondrogenic differentiation and persistent proliferation of chondrosarcomas. In line with this, in chondrosarcoma cell lines miR-129-5p repressed WNT/β-catenin signaling by targeting *SOX4* which repressed proliferation and invasion [41].

Also, adult AC contains MSPC expressing MSC related markers [42], which are predominantly localized in the superficial zone (SZ) [43,44] and undergo proliferation upon onset of OA [44]. Depending on the study, the human AC MSPC population was defined as positive for CD105 and CD166 [45,46,47], STRO-1 [48], NOTCH1 [49], CD166 and CD90 [50], STRO-1 and FGF2 [51] or CD106, STRO-1 and NOTCH1 [43,49]. The MSPC fraction makes up 3–17% of all AC resident cells and increases in human OA AC compared to normal adult AC [46,47,49,52]. Utilizing a colony-forming assay, Fellows et al. reported a doubling of the MSPC population in human OA AC compared to normal adult AC [53]. Moreover, it seems that especially OA AC contains two MSPC populations. One population consists of more committed cartilage progenitor cells exhibiting a limited proliferation potential and early senescence, which may either arise from dedifferentiated chondrocytes or activated cartilage inherent quiescent progenitors. A second population consists of rather multipotent stem cells, which are either inherent, since they are also found in normal adult AC, or which may be also recruited from adjacent tissues like bone marrow or synovium [53]. Whether the increase of MSPC number in OA AC is an attempt of cartilage intrinsic repair or rather a prerequisite for macroscopic cartilage degradation due to a lack of extracellular matrix (ECM) maintenance, respectively proliferation-associated degradation, remains elusive. 

Culturing of human bone marrow-derived MSPC with rFGF2 reduced the cell size and turned the cell shape into a spindle-like fibroblastic-like appearance, which was accompanied by a faster growth, increased life span and an advance in chondrogenic potential [54,55,56,57]. FGF2 signaling was mediated by fibroblast growth factor receptor 1 (FGFR1) activity, which was rate limiting for self-renewal of human MSPC [58]. Interestingly, telomere length of MSPC expanded under rFGF2 increased. Since no telomerase activity was detected, FGF2 seems to selectively expand a subpopulation of cells with longer telomeres [59]. Moreover, in human MSPC FGF2 increased SOX5, SOX6 and SOX9 expression, which are all implicated in enhanced chondrogenesis [60], although siRNA-mediated knockdown of *SOX9* did not prevent rFGF2-mediated chondrogenesis [57]. Notably, aging of human bone marrow derived MSPC progressively decreased FGF2 and FGFR1 expression, which coincided with reduced proliferative capacity of MSPC after prolonged cultivation [61]. 

Interestingly, around the age of 40, when chondrosarcoma incidence starts to rise, also MSPC in the SZ of AC start to proliferate and exhibit reorganization of cell arrangement which may be a prerequisite or early indicator of OA [62,63]. Collectively, this indicates a proliferation-inducing environment in cartilage and bone at that age in which increasing abundance of free FGF2 may be implicated. 

## 3. Hypoxia and Angiogenesis in the Bone Niche

In the bone, chondrosarcoma cells interact with resident MSPC, HSC, mature immune cells, (pre)osteoblasts, (pre)osteoclasts and endothelial cells [21]. Chondrosarcoma growth dysregulates the balance between osteoblasts and osteoclasts leading to bone degradation, which might be mediated by increased RANKL expression by chondrosarcoma cells [64]. 

Without neovascularization the size of a tumor is inherently limited to 1–5 mm due to restricted oxygen and nutrient availability [65]. Although conventional chondrosarcomas remain poorly vascularized compared to other tumors [66,67], which may contribute to the cartilage like differentiation and systemic chemotherapy resistance, induction of angiogenesis is the major limiting step towards local invasion and metastasis. This is reflected by a low rate of vascularization in G1 conventional chondrosarcomas, which is largely enhanced in the significantly more aggressive G2 and G3 chondrosarcomas [66,68]. VEGF and FGF2, which have an intense crosstalk, are the two main growth factors involved in chondrosarcoma angiogenesis [65]. VEGF, FGF2, endothelin-1 (ET-1) and hypoxia-inducible factor-1α (HIF-1α) expression and mitogen activated protein kinase (MAPK) signaling are significantly enhanced in G2 and G3 conventional chondrosarcomas compared to G1 chondrosarcomas [65,68,69,70,71,72]. In addition, central chondrosarcomas commonly express FGFR1, whereas FGFR3 expression is rarely detected [13]. Moreover, high HIF-1α expression has been linked to shorter disease free and overall survival in chondrosarcoma patients [73,74]. 

Interestingly, dedifferentiated chondrosarcomas showed a high microvessel density without correlation to the grade of the tumor [67] or VEGF expression [70]. FGF2 exerts its pro-angiogenetic activities by interaction with a variety of surface receptors on endothelial cells including receptor tyrosine kinases, heparan-sulfate proteoglycans and integrins. Subsequently, endothelial cells upregulate VEGF, FGF2, OPN and cyclooxygenase 2 (COX-2) expression. Moreover, FGF2 induces the expression of inflammatory cytokines and acts as a direct chemoattractant for immune cells, which actively participate in neovascularization of various tumors [75]. C-X-C motif chemokine ligand 12 (CXCL12) is secreted in the bone marrow, where it mediates the homing of HSC [76]. C-X-C motif chemokine receptor 4 (CXCR4) is the receptor for CXCL12, which is typically expressed by HSC and mature immune cells. Both expression of CXCL12 and CXCR4 can be upregulated by HIF-1α under hypoxic conditions in several cell types [76]. Moreover, CXCR4 downstream signaling has been implicated in migration and metastasis of different tumors [76]. Notably, both CXCR4 and CXCL12 expression was increased in human chondrosarcoma tissues [77] and CXCR4 expression was also enhanced by hypoxia induced HIF-1α and VEGF-A expression in chondrosarcoma cell lines concomitantly with MMP1 expression [78,79,80], whereas CXCL12 induced VEGF-A secretion under hypoxic conditions [80], indicating a self-reinforcing system. In addition, miR-181a was overexpressed in high grade chondrosarcomas and its expression was upregulated by hypoxia in human chondrosarcoma cell lines. Regulator of G-protein signaling 16 *(RGS16)*, a negative regulator of CXCR4 signaling is a direct target of miR-181a [78], implicating this miRNA in activation of CXCR4 signaling. C-C motif chemokine ligand (CCL5), also known as regulated upon activation, normally T-expressed, and presumably secreted (RANTES) has been implicated in downregulation of miR-199a in human chondrosarcoma cells, which promotes VEGF upregulation and angiogenesis [81]. In vitro, rFGF2 treated chondrosarcoma cells increased VEGF-C expression via downregulation of miR-381 [82]. In addition, CCN2, also known as connective tissue growth factor (CTGF), has been identified as a binding partner of VEGF-A (Figure 2). In complex with rCCN2, rVEGF-A was not able to induce angiogenesis in a human in vitro system as well as in vivo in mice [83]. The CCN2-VEGF-A complex could be dissociated by CCN2 cleavage by MMP-1, MMP-3, MMP-7 and MMP-13 reactivating the angiogenetic activity of VEGF-A in vitro and in a matrigel injection model in mice [84]. Indeed, in high grade chondrosarcomas *CCN2* mRNA was downregulated compared to low grade chondrosarcomas [85]. In contrast, rCCN6 promoted angiogenesis in human chondrosarcoma cell lines SW1353 and JJ012 by induction of VEGF-A expression through inhibition of miR-452, which interacts with the 3′UTR of the human VEGF-A gene repressing its transcription [86]. 

Notably, Wang et al. showed that human growth plate cartilage expressed the NH2-propeptide of the cartilage-characteristic collagen type IIB splice variant (PIIBNP), which was capable of killing both human chondrosarcoma and carcinoma cells upon binding of αvβ3 and αvβ5 integrins in vitro [87]. These integrin types were not expressed on cells of human developing cartilage, whereas adult human AC cells expressed low levels [88]. In contrast, human osteoclasts and endothelial cells express high levels of αvβ3 and αvβ5 integrins [89,90], indicating that they are actively excluded from the growth plate. Whether PIIBNP is also present in adult healthy or OA AC has not been investigated.

In summary, vascularization is an important step during chondrosarcoma progression and appears to be predominantly mediated by VEGF and FGF2. Whereas avascularity of developing cartilage seems to be ensured by active exclusion of endothelial cells.

## 4. Local Invasion and Metastasis

Catabolic degradation of the ECM, an important prerequisite for migration and tumor cell invasion, is predominantly mediated by MMP [91]. Basically, MMP expression is a stem cell feature [92], but also differentiated cells may upregulate MMP expression upon senescence [93] or inflammation [94]. MMP-1, MMP-2, MMP-3, MMP-7, MMP-9 and MMP-13 are frequently expressed in chondrosarcoma tissues [95,96]. In addition, high levels of MMP-26 were detected in chondrosarcoma samples by Xu et al. [97]. Increased MMP-1 expression has been linked to poor outcome in chondrosarcoma patients [95] and also promotes invasiveness of human chondrosarcoma cells in vitro [98]. A single nucleotide polymorphism (SNP) frequently found in the promoter region of MMP-1 in human chondrosarcoma tissues and established chondrosarcoma cell lines [99] generates an artificial avian erythroblastosis virus E26 oncogene homolog (ETS) binding site, which has been implicated in enhanced MMP-1 expression in several carcinomas [100]. Especially in high grade central chondrosarcomas increased MMP-2 levels have been documented [101,102]. But also expression of MMP-3, MMP-7 and MMP-13 increased with tumor grade [103]. Often, MMP expression was highly variable in different areas of a single chondrosarcoma [104]. Moreover, MMP-1, MMP-7 and MMP-9 expression was found to be highest in the invasive protrusions of chondrosarcomas [95,103]. 

Also, elevated expression of pro-inflammatory CCL5, CCL3 and their receptor C-C motif chemokine receptor 5 (CCR5), which is a typical surface protein of macrophages and T-cells, has been detected in human chondrosarcoma tissues (Figure 2) [105]. In vitro, rCCL3 induced MMP-2 expression and migration in the JJ012 chondrosarcoma cell line, which was mediated by AMP-activated protein kinase (AMPK), p38 MAPK and nuclear factor kappa B (NF-κB) [102]. rCCL5 induced MMP-3 expression and migration via phosphatidylinositol 3-kinase (PI3K), v-Akt murine thymoma viral oncogene homolog (AKT) and NF-κB in the same chondrosarcoma cell line [105]. 

Adipose tissue may function as endocrine organ by secretion of adipokines including pro-inflammatory cytokines [106]. Resistin, also known as adipose tissue-specific secretory factor (ADSF), has been identified as peptide hormone secreted by murine adipose tissue. Notably, in humans especially, monocytes express resistin [107]. High resistin levels in human chondrosarcoma tissues have been linked to MMP-2 expression and r-resistin promoted invasiveness and MMP-2 expression in human chondrosarcoma cells in vitro mediated by AMPK and p38 MAPK [108]. Another adipokine implicated in chondrosarcoma migration and angiogenesis is adiponectin, which is increasingly upregulated with the histological grade of conventional chondrosarcoma [109]. In addition, elevated expression of the two adiponectin receptors *ADIPOR1* and *ADIPOR 2* mRNA was detected in human chondrosarcoma tissues and cell lines [110]. In chondrosarcoma cell lines, r-adiponectin promoted VEGF-A expression via ADIPOR activating the PI3K-AKT-mTOR pathway and HIF-1α [109]. Migration was induced via ADIPOR activating AMPK, p38 and NF-κB pathways [110].

ET-1 is a peptide hormone secreted by vascular endothelial cells and monocytes [111]. COX-2, a key enzyme in prostaglandin biosynthesis, mediates inflammation, angiogenesis and cancer progression [112]. Significant ET-1 and COX-2 expression in human chondrosarcoma tissues has been linked to chondrosarcoma cell migration in vitro. In JJ012 cells rET-1 increased COX-2 expression via ET-receptors, MAPK, and activator protein 1 (AP-1) signaling.

OPN is secreted by bone cells like osteoblasts and osteoclasts, but also immune cells and endothelial cells [113]. It demonstrably promotes progression of different cancers by enhancing proliferation, survival, angiogenesis, motility, and invasion. In the bone, its main functions are the regulation of mineralization and remodeling [113]. The expression of OPN can be upregulated by different growth factors including FGF2 in murine endothelial cells [114,115], whereas OPN is involved in COX-2 and HIF-1α dependent VEGF expression in human carcinoma and melanoma [114]. rOPN increased MMP-9 expression and migration of JJ012 chondrosarcoma cells through αvβ3 integrin, focal adhesion kinase (FAK), MAPK/ERK kinase (MEK), extracellular signal-regulated kinase (ERK) and NF-κB signaling [116].

In human HCS-2/8 chondrosarcoma cells rFGF2 induced MMP-13 expression [72]; rCCN2, which interacts both with FGF2 and FGFR, increased MMP-13 expression and migration in human JJ012 cells in vitro via αvβ3 integrin, FAK, ERK, and NF-κB [117]. In addition, rMMP-3 has been shown to upregulate the expression of CCN2 in the human chondrosarcoma cell line HCS-2/8 in vitro [118]. However, in human high-grade chondrosarcoma tissues *CCN2* mRNA was reduced compared to low grade chondrosarcomas [85]. rCCN6 enhanced migration of the human chondrosarcoma cell line JJ012 by increasing intercellular adhesion molecule 1 (ICAM-1) expression via αvβ3 and αvβ3 integrins, FAK and MAPK signaling [119]. 

Collectively, these data indicate that the signaling events that induce MMP-based ECM degradation and local invasion are closely interconnected to those that mediate inflammation and angiogenesis. Yet, metastasis can only occur when cancer cells are able to settle down and proliferate in a foreign environment [120]. For chondrosarcoma, the lung is the primary site of metastasis [5], albeit further research is needed to elucidate the reasons for this and how it may be prevented.

## 5. Primary Cilia in Chondrogenesis, Cartilage Maintenance and Chondrosarcoma

In mammals, primary cilia mediate cellular responses to mechanical load but also substrate texture by activation of different signaling pathways including wingless-type MMTV integration site family (WNT) and hedgehog (Hh) [121,122,123]. Loss of primary cilia in human MSPC inhibited adipogenic and osteogenic differentiation to a greater extent than chondrogenic differentiation (Figure 1) [124]. Nevertheless, chondrogenesis of human MSPC can be induced by dynamic compression of 3D cultures [125,126,127], which has to be perceived by the cells through an appropriate mechanism which may be localized in the primary cilia. Interestingly, MSPC in the SZ of human OA AC seem to have more and longer cilia compared to cells in healthy AC [128]. This difference in cilia length has been also demonstrated in the SZ of bovine AC [129]. In human MSPC increased cilia length has been associated with decreased WNT signaling and decelerated proliferation [123]. Indeed, primary cilia claim the basal body and therefore the centrosome for assembly, which means that primary cilia have to be disassembled for the formation of the mitotic spindle, which takes more time the longer the cilium is [130]. On the other hand, fast growing cells may not have the time to assemble primary cilia between cell divisions. In tumor cells, both the lack of primary cilia but also formation of multiple cilia due to inhibited cytokinesis in multinucleated cells with several centrosomes has been described [131]. Human chondrosarcoma cells lose primary cilia with increasing malignity [132], which may be either a consequence of rapid proliferation or the prerequisite for enhanced cell cycle progression. Recently, histone deacetylase 6 (HDAC6) has been implicated in suppression of cilia formation in human chondrosarcoma [133]. Notably, osteochondroma have a random cilia orientation compared to normal growth plate chondrocytes where cilia are arranged parallel to the growth axis, which assures the correct assembly of chondrocytes in stacked columns [134]. Upon closure of the growth plate usually also osteochondroma growth stops [135]. However, resting MSPC in the metaphysis of long bones may be reactivated during formation of secondary chondrosarcoma [14]. In summary, the progressive loss of primary cilia in conventional chondrosarcomas may foster chondrogenic differentiation, but also facilitate proliferation.

## 6. Chondrosarcoma Treatment

### 6.1. State of the Art

The risk of local recurrence and metastasis of conventional chondrosarcomas largely increases with histological grade [6]. For low grade tumors intralesional excision may be sufficient [136], whereas surgery with wide margins has become the primary care for malignant cartilage lesions [137]. Yet, depending on the location in the skeleton, wide resection may not be possible [138,139] and adjuvant therapies may be needed. Unfortunately, conventional chondrosarcomas are highly resistant both to radiation and chemotherapy [139,140,141]. Chemoresistance of chondrosarcoma may be due to slow proliferation, multidrug resistance protein 1 (MDR1) overexpression [142,143], poor vascularity [66,67] and dense hyaline ECM [144] when compared to other cancers. Notably, under moderate hypoxic conditions (5% O_2_) radiation resistance of chondrosarcoma cell lines was significantly higher compared to standard cell culture conditions (21% O_2_) [141], indicating that prevalent hypoxia may also interfere with radiation response in chondrosarcoma. Yet, there are publications indicating sustained stable disease after chemotherapy in palliative care for some patients with dedifferentiated chondrosarcoma [8,145,146,147]. Also, proton beam radiation therapy resulted in sustained local control of some chondrosarcomas of the skull base [148]. Besides, carbon ion radiation may be useful for inoperable pelvic chondrosarcomas [149].

In addition, chondrosarcomas tend to be genetically instable with loss of heterozygosity at many loci [150,151], which exacerbates identification of relevant targets and raises the question whether single agent approaches may be applicable at all. Moreover, many older studies investigating genetic aberrations and potential target proteins in chondrosarcoma did not distinguish between different subtypes and gradings.

In summary, there is an urgent need for new targeted therapies in chondrosarcoma which are discussed in the following paragraphs.

### 6.2. Targetting the Hh Pathway

The Hh pathway is involved in stem cell proliferation and differentiation during development and tissue regeneration [152]. In addition, Hh signaling is also implicated in the formation and progression of several kinds of cancer, including basal cell carcinoma, medulloblastoma, osteosarcoma, Ewing sarcoma and rhabdomyosarcoma [153,154,155,156,157]. The Hh pathway is activated by binding of one of the three human ligands (IHH, DHH, SHH) to the patched 1 (PTCH1) receptor. Subsequently, smoothened (SMO) initiates downstream signaling via the glioma associated oncogene family (GLI) transcription factors [158]. Depending on the tumor, aberrant Hh pathway activation may be mediated by ligand overexpression or PTCH loss-of-function mutations. But also ligand-independent activation via SMO gain-of-function mutations, respectively constitutive overexpression and activation of GLIs is possible [154]. Yan et al., 2008 detected a single SMO mutation in a selection of dedifferentiated chondrosarcomas. All SNPs detected in the PTCH1 gene of chondrosarcomas resulted in silent alterations [159]. Therefore, mutations in the Hh pathway seem to be infrequent in chondrosarcomas. However, Tiet et al., 2006 reported a constitutive active Hh pathway in human chondrosarcoma explant cultures similar to growth plate chondrocytes, although in high grade chondrosarcomas IHH, PTCH1 and GLI1 mRNA expression declined when compared to low grade lesions [160,161]. Also in peripheral chondrosarcomas PTCH1, GLI1 and GLI2 mRNA expression was reduced compared to benign osteochondromas [162].

Indeed, in animal models ligand dependent Hh signaling required primary cilia [122]. Therefore, drug efficacy of SMO inhibitors like vismodegib (GDC-0449) or saridegib (IPI-926) is probably dependent on the presence of primary cilia, where Hh pathway components have been shown to be concentrated in murine chondrocytes [163]. Yet, Ho et al. observed that most chondrosarcoma and enchondroma cells (70–100%) lack primary cilia, whereas 65% of human AC chondrocytes had primary cilia [132]. Notably, ablation of primary cilia in chondrosarcoma explants enhanced Hh pathway regulated transcription, probably via prevention of GLI3 processing in its repressor form, whereas the SMO inhibitor cyclopamine was ineffective in these cells [132]. 

With this in mind, it is not surprising that GDC-0449 did not meet the primary endpoint in a phase II clinical trial with chondrosarcoma patients, although some patients with grade I or II chondrosarcomas seemed to benefit from the treatment [164]. Also the results of a phase II trial using IPI-926 for treatment of chondrosarcoma patients were discouraging [5].

Potentially, the use of Hh pathway inhibitors acting downstream of SMO like HPI-4 may be more successful in chondrosarcoma treatment by means of targeting cancer stem cells and early grade chondrosarcoma cells [165], but maybe also later stages as its mechanism of action is not necessarily dependent on the presence of primary cilia. In the SW1353 chondrosarcoma cell line the use of arsenic trioxide (ATO) induced G2/M cell cycle arrest and apoptosis as well as autophagy. In addition to the GLI transcription factors ATO has several other targets and indeed, autophagy induction in SW1353 cells was reported to depend on mTOR inhibition [166]. Thus, using ATO alone or in combination with other substances may aid to develop novel treatment strategies for chondrosarcomas.

### 6.3. Targetting IDH1, IDH2 and HDACs

The isocitrate dehydrogenases 1 and 2 (IDH1 and IDH2) catalyze the conversion of isocitrate to to α-ketoglutarate (α-KG) in the Krebs cycle and concomitantly produce nicotinamide adenine dinucleotide phosphate (NADPH) from NADP+ [167]. Missense mutations at arginine 132 in IDH1 or the homologous arginine 172 in IDH2 occur in 80–90% of enchondromas [168,169] and 50–60% of central chondrosarcomas with IDH1 mutations dominating [170]. Also, dedifferentiated chondrosarcomas predominantly contain IDH1 mutations [171]. Indeed, IDH mutations seem to be an early event in chondrosarcoma genesis [171]. The mutant enzymes have a modified enzymatic activity reducing α-KG under consumption of NADPH to the onco-metabolite R(−)-2-hydroyglutarate (2-HG) [167]. IDH mutations are always restricted to one allele and it seems that the presence of the wild type protein in a homodimer increases the capacity of 2-HG generation [172]. Many cellular enzymes are dependent on the presence of α-KG including the Ten-Eleven-Translocation (TET) protein family reducing DNA methylation, the JumonjiC domain-containing (JmjC) histone demethylases altering histone methylation, prolyl and lysyl hydroxylases implicated in collagen folding and maturation, and prolyl hydroxylases (PHD) regulating hypoxia-inducible factor (HIF) protein degradation [172]. Thus, mutant IDH is capable of altering the epigenetic state of cells leading to DNA and histone hypermethylation which affects differentiation [173] and impairs collagen maturation as well as oxygen homeostasis [174]. Indeed, generation of 2-HG not only reduces α-KG abundance but 2-HG directly inhibits enzymes dependent on α-KG by occupying the binding site [172]. 

In human MSPC, the expression of the IDH1 R132C mutant also upregulated global histone methylation, but repressed osteogenic differentiation and induced chondrogenic differentiation (Figure 1) indicated by SOX9 and collagen type II α 1 chain (COL2A1) expression, whereas no functional cartilage matrix was deposited [175]. This effect might contribute to the incomplete chondrogenic differentiation of chondrosarcomas. 

Inhibition of the mutant IDH1 in the human chondrosarcoma cell line JJ012 (IDH1 R132G) by AGI-5198 significantly reduced 2-HG production, colony formation and migration and induced apoptosis [176], illustrating that IDH1 inhibition may have therapeutic value. 

Indeed, different clinical studies targeting IDH mutant chondrosarcomas and other tumors are currently ongoing. The NCT02273739 phase I/II, multicenter study has been actually completed. In this study, AG-221, an oral IDH2 inhibitor, has been tested in patients with advanced solid tumors, including gliomas, angioimmunoblastic T-cell lymphomas and chondrosarcomas with an IDH2 mutation. In two other ongoing phase I studies (NCT02481154/NCT02073994), the IDH inhibitors AG-881 and AG-120 are under clinical evaluation for advanced solid tumors that harbor an IDH1 and/or IDH2 mutation, including gliomas, cholangiocarcinomas and chondrosarcomas. In addition, there is a phase Ib, open-label, single-center, nonrandomized study recruiting patients that evaluates the toxicity and efficacy of the antidiabetic drug metformin in combination with the antimalarial drug chloroquine in IDH1/2 mutated patients with a glioma, intrahepatic cholangiocarcinoma or chondrosarcoma (NCT02496741) [177]. 

In addition to methylation, acetylation of histones regulates gene expression and differentiation. Since many tumors exhibit aberrant histone modifications, HDAC inhibitors have emerged as new class of anticancer drugs [178]. Indeed, the HDAC inhibitor depsipeptide (romidepsin) induced growth arrest, apoptosis and differentiation in human chondrosarcoma cell lines in vitro [179]. A phase II study (NCT00112463) including extra-skeletal chondrosarcoma patients treated with romidepsin has been actually completed. Whether IDH inhibition or HDAC inhibition is indeed a beneficial therapy in chondrosarcoma remains to be demonstrated.

In line with chondrosarcomas, accumulating evidence also shows epigenetic dysregulation in OA [180,181]. However, IDH mutations have not been detected in AC so far, but inflammatory cytokines suppressed IDH and TET activity in human primary chondrocytes in vitro [182], a mechanism which might also apply in chondrosarcomas without IDH mutation.

### 6.4. Tagetting the PI3K-AKT-mTOR and SRC Pathway

AKT kinases are highly active in human chondrosarcomas [183,184]. Indeed, PI3K-AKT signaling is activated by many growth factors including FGF2 and IGF1 as well as inflammatory cytokines like CCL5 (Figure 2), which have been implicated in chondrosarcoma genesis and progression [185]. In addition, p70 S6 kinase (p70S6K) activation was increased with histological grade of conventional chondrosarcomas and has been also detected in dedifferentiated chondrosarcomas [186]. P70S6K is a downstream target of mTOR in the PI3K-AKT pathway phosphorylating the ribosomal S6 protein, which enhances protein synthesis [5]. However, activating mutations in the PI3K-AKT pathway are very rare in chondrosarcomas [187]. Although IGF1R positivity of human central chondrosarcomas increases with histological grade, IGF1R inhibition did not impair proliferation or migration of chondrosarcoma cell lines in vitro [188]. Treatment with BEZ235, a PI3K/mTOR inhibitor, significantly reduced growth of chondrosarcoma cell lines in vitro and in a murine xenograft model [186]. In a rat chondrosarcoma model, the use of the mTOR inhibitor everolimus suppressed tumor progression and delayed recurrence of microscopic residual disease [189]. Moreover, in a small retrospective study with patients with unresectable chondrosarcoma a combination of sirolimus (rapamycin) inhibiting mTOR and the chemotherapeutic agent cyclophosphamide was successful in disease control in 70% of patients during a period of several months [190]. Yet, a phase II clinical trial (NCT02008019) utilizing everolimus in patients with primary or relapsed chondrosarcomas has been suspended in 2016 due to unavailability of everolimus.

Aberrant activation of SRC kinase signaling has been detected in human chondrosarcoma tissues [183,191]. SRC is a common mediator of growth factor and integrin signaling and may also act upstream of PI3K-AKT [185]. Together SRC and AKT activate survival pathways and HIF1α, which is upregulated in high grade chondrosarcoma tissues [73,74]. 

Dasatinib (BMS354825), which targets SRC as well as Abelson tyrosine protein kinase (ABL), KIT and platelet derived growth factor receptor (PDGFR) decreased viability of chondrosarcoma cell lines in vitro [183,192]. Moreover, dasatinib especially sensitized p53 mutant chondrosarcoma cell lines to doxorubicin treatment, indicating a potential of dasatinib to overcome chemoresistance [191]. Nevertheless, in the SRC009 phase II trial, the use of dasatinib as single agent in pretreated, high-grade sarcomas, including chondrosarcomas showed no benefit [193]. Therefore, inhibition of PI3K-AKT-mTOR and SRC might be advantageous in combination therapy of chondrosarcoma patients, although reliable clinical data are still missing.

### 6.5. Targeting Angiogensis and Invasion

As already discussed in chapter 3 and 4, both VEGF and FGF2 signaling are activated in chondrosarcomas and apparently contribute to angiogenesis and invasion. Since enhanced activation of PDGFR has been shown in human chondrosarcoma cell lines in vitro [183], PDGFR may be a therapeutic target as well. SU6668, an inhibitor of vascular endothelial growth factor receptor 2 (VEGFR2), PDGFR-β and FGFR1 induced growth inhibition in chondrosarcoma animal models, which seems to be attributed to the antiangiogenic effects of SU6668 [194]. The ongoing NCT01330966 phase II study is investigating the efficacy and safety of the single agent pazopanib, inhibiting KIT, FGFR, PDGFR and VEGFR among other enzymes [195], in patients with unresectable or metastatic chondrosarcoma. Another phase II trial (NCT02389244) currently recruiting patients utilizes regorafenib, an oral multikinase inhibitor, which targets VEGFR, tyrosine kinase with Ig and EGF homology domains-2 (TIE2), KIT, rearranged during transfection (RET), RAF-1, BRAF, BRAFV600E, PDGFR and FGFR, in patients with metastatic bone sarcomas including chondrosarcomas. Sorafenib, a multi-kinase inhibitor inhibiting several tyrosine kinases including VEGFR, PDGFR and RAF, mediated pMEK and pERK inhibition leading to growth arrest and apoptosis in the chondrosarcoma cell lines SW1353 and CRL7891, which was accompanied by downregulation of cyclin D1, retinoblastoma suspectibility protein (RB), B-cell lymphoma-extra large (BCL-XL) and myeloid cell leukemia sequence 1 (MCL-1) expression [196]. Two phase II studies utilizing sorafenib in patients with different types of sarcomas indicated prolonged stable disease when evaluated in 3 chondrosarcoma patients [197,198]. Moreover, imatinib, another multi-kinase inhibitor, inhibiting ABL, KIT and PDGFR, was tested in a phase II trial with patients having recurrent, non-resectable, PDGFR positive chondrosarcomas. However, in this trial no benefit of imatinib treatment has been reported [199]. Another open-label study (NCT00928525) utilizing imatinib in chondrosarcoma patients is ongoing. Once data of the ongoing trials are available, it may be determined, whether the use of multikinase inhibitors is a therapeutic option for chondrosarcoma patients. 

### 6.6. Additional Targets and Biomarker

In addition to the pathways mentioned in the previous chapters that are currently being investigated in clinical trials, there may be other signaling pathways involved in chondrosarcoma genesis and progression that could serve as potential novel targets.

Canonical WNT signaling is implicated in the β-catenin-dependent regulation of mitotic and cell fate-determining gene transcription of MSPC, whereas two non-canonical WNT pathways affect cell shape and motility in the planar cell polarity pathway and the Ca^2+^/WNT pathway [200]. Dickkopf WNT signaling pathway inhibitor 1 (DDK1), an antagonist of canonical WNT/β-catenin signaling as well as β-catenin were progressively overexpressed in chondrosarcoma tissues with increasing histological grade and correlated with poor prognosis [201]. In the chondrosarcoma cell line SW1353 *WNT3A*, *WNT6*, *WNT7B* and frizzled-3 *(FZD3)* mRNA expression was upregulated compared to MSPC and rWNT3A enhanced SW1353 proliferation [202]. Recombinant WNT inhibitory factor 1 (WIF1), which inhibits WNT signaling by binding of several WNT ligands including WNT3A and WNT5A, prevented WNT induced MSPC growth by neutralizing rWNT3A-mediated inhibition of chondrogenesis in micromass cultures of embryonic chick limb bud cells [203]. WIF1 is epigenetically silenced via promoter methylation in human chondrosarcoma tissues and cell lines, and loss of WIF1 protein expression correlated with lower progression free and overall survival rates [204]. Interestingly, aging reduced expression of β-catenin in bone marrow derived MSPC, while β-catenin phosphorylation increased, indicating enhanced proteasomal degradation [61]. Therefore, targeting of WNT signaling might be of interest for chondrosarcoma treatment. 

NOTCH signaling maintains the stem cell phenotype and prevents differentiation of different types of MSPC. In 3D cultures of hMSPC NOTCH signaling was downregulated with increased chondrogenic differentiation. Indeed, jagged 1 (JAG1) overexpression prevented chondrogenesis in hMSPC [60]. In adult AC NOTCH1 expression is restricted to MSPC in the SZ which proliferate during the onset of OA and form clusters [49]. In a human conventional chondrosarcoma of the maxilla strong NOTCH3 and JAG1 protein expression was detected at areas of tumor proliferation [205]. Increased NOTCH1, hairy and enhancer of split 1 (HES1), hairy/enhancer-of-split related with YRPW motif (HEY) 1 and HEY2 protein expression indicating active NOTCH signaling has been detected in human chondrosarcoma tissues [206]. In conclusion, inhibition of NOTCH signaling might impede proliferation and induce differentiation in chondrosarcomas.

Intriguingly, 96% of high grade central chondrosarcomas harbor alterations in the p53 or RB tumor suppressor pathways [207]. Inactivating p53 mutations especially occur in G3 chondrosarcomas [2], indicating a rather late event. In addition, amplification of 12q13 and loss of 9p21 are common genetic changes in advanced chondrosarcomas [135]. Indeed, the genes for mouse double minute 2 (*MDM2*), negatively regulating p53, and cyclin dependent kinase 4 (*CDK4*), important for G1 cell cycle progression, are localized at 12q13, whereas 9p21 contains the cyclin dependent kinase inhibitor 2A (*CDKN2A*) locus, coding for the tumor suppressor proteins p16INK4A and alternative reading frame (ARF) [208,209]. Lack of p16INK4A was implicated in the radiation resistance of human chondrosarcoma cell lines in vitro [141]. Although the loss of these tumor suppressor genes is frequently found in advanced chondrosarcomas, their inactivation might be also a result of increasing genomic instability [150,151] and not the primary cause of tumor progression.

Programmed cell death ligand 1 (PD-L1) expression, either by tumor cells themselves or by the tumor-associated immune cells, can be an effective mechanism for tumors to evade T-cell recognition and destruction [210]. Indeed, 41% of dedifferentiated chondrosarcomas examined in a study of Kostine et al. displayed PD-L1 positivity. Preliminary results of the SARC028 phase II study utilizing the anti-PD1 antibody pembrolizumab (MK-3475) in patients with advanced soft tissue and bone sarcomas showed in one out of six dedifferentiated chondrosarcoma patients a partial tumor remission [5] indicating a potential use of this therapy in a subset of chondrosarcoma patients.

As already discussed in the previous chapters, several miRNAs are dysregulated in their expression in human chondrosarcomas [211]. However, functional implication in chondrosarcoma tumorigensis has yet only been attributed to a few of them, although many have distinct functions in other types of cancer. Depletion of two miRNAs has been implicated in chemoresistance; miR-100 is downregulated in human chondrosarcoma tissues and cell lines. In vitro, depletion of miR-100 resulted in cisplatin resistance of chondrosarcoma cells probably mediated by mTOR, which is a direct target of miR-100 [212]. Low miR-125b level have been associated with doxorubicin resistance of chondrosarcoma cells in vitro [213]. In the human chondrosarcoma cell line JJ012 predominantly NOTCH and IGF signaling were deregulated by reduced endogenous miRNA levels [214]. Hence, the interest in miRNAs in cancer is consistently growing. 

Although some individual targets seem to be promising for chondrosarcoma treatment, in the clinic, the use of single targeted drugs just like chemotherapeutics and radiation is often disappointing. This might be due to global epigenetic and genomic changes in chondrosarcoma, but also some special features including poor vascularity and dense ECM accompanied by permanent activation of stem cell pathways preventing differentiation. In addition, the rarity of chondosarcomas prevents initiation of large stratified trials. 

## 7. Conclusions

Chondrosarcomas are a heterogenic group of rare tumors. Basically, some entities seem to arise from MSPC of the growth plate in adolescents and young adults. On the other hand, quite unusual for bone sarcomas, the peak incidence of conventional and dedifferentiated chondrosacomas is in adults beyond the age of 40. Indeed, these chondrosarcomas seem to originate from MSPC in the bone marrow, but not necessarily in the long bones. High grade conventional, but especially dedifferentiated chondrosarcomas have an irregular appearance, with zones of rather chondrogenic and areas of rather fibrogenic or osteogenic differentiation, that together indicate conflicting signaling, or even a heterogenous pool of MSPC, which prevents differentiation in one or the other direction. Cartilaginous differentiation is a challenge for tumor growth, as avascularity—a hallmark of cartilage—limits the growth rate. Actually, low grade conventional chondrosarcomas do slowly grow and are rather benign. However, once vascularization is induced, conventional and dedifferentiated chondrosarcomas tend to be locally aggressive and also frequently metastasize to the lung. In addition, high grade conventional chondrosarcomas and dedifferentiated chondrosarcomas exhibit a remarkable therapeutic resistance comprising chemotherapy, radiation therapy, but also targeted approaches. This has been attributed to comparatively slow proliferation, MDR1 overexpression, relatively poor vascularization and a dense hyaline ECM. Sustained stemness as well as global epigenetic and genomic changes seem to be implicated in therapeutic resistance of chondrosarcomas. In addition to the challenge to identify relevant targets for this rare disease, single agent approaches may be not applicable at all. Due to various subtypes and grading specific differences, identification of biomarkers for stratification of chondrosarcoma patients is urgently needed. Actually, clinical trials targeting mutant IDH, HDAC, PI3K/AKT/mTOR and SRC signaling as well as FGF2, VEGF and PDGF pathways are ongoing and have to be evaluated. Furthermore, immunotherapy has to be considered as a therapeutic option. 

Notably, at the age beyond 40 at which the conventional and dedifferentiated chondrosarcoma incidence starts to rise, also MSPC in the SZ of AC start to proliferate and exhibit reorganization of cell arrangement which may be a prerequisite or early indicator of OA. Collectively, this indicates a changed microenvironment in cartilage and bone, in which an increasing abundance of free FGF2 might be implicated, since FGF2 signaling is involved in proliferation, migration, inflammation and angiogenesis. To date, it has not been investigated whether chondrosarcoma patients may additionally suffer from progressive OA or obesity. Such data may help to answer whether both diseases induce a growth factor and cytokine milieu that is supportive of chondrosarcoma growth and progression once chondrosarcoma-specific genetic and epigenetic aberrations have occurred in bone marrow MSPC.

## Abbreviations

2-HG	R(−)-2-hydroyglutarate
α-KG	α-ketoglutarate
ABL	Abelson tyrosine protein kinase
AC	articular cartilage
ADIPOR	adiponectin receptor
ADSF	adipose tissue-specific secretory factor
AKT	v-Akt murine thymoma viral oncogene homolog
AMPK	AMP-activated protein kinase
AP-1	activator protein 1
ARF	alternative reading frame
ATO	arsenic trioxide
BCL-XL	B-cell lymphoma-extra large
CCL	C-C motif chemokine ligand
CCR	C-C motif chemokine receptor
CD	cluster of differentiation
CDK4	cyclin dependent kinase 4
CDKN2A	cyclin dependent kinase inhibitor 2A
COL2A1	collagen type II α 1 chain
COX-2	cyclooxygenase 2
CTGF	connective tissue growth factor
CXCL12	C-X-C motif chemokine ligand 12
CXCR4	C-X-C motif chemokine receptor 4
DHH	desert hedgehog
DDK1	dickkopf WNT signaling pathway inhibitor 1
ECM	extracellular matrix
ERK	extracellular signal-regulated kinase
ET-1	endothelin-1
ETS	avian erythroblastosis virus E26 oncogene homolog
FAK	focal adhesion kinase
FGF2	fibroblast growth factor
FGFR1	fibroblast growth factor receptor 1
FZD3	frizzled-3
GLI	glioma associated oncogene family
HDAC6	histone deacetylase 6
HES1	hairy and enhancer of split 1
HEY	hairy/enhancer-of-split related with YRPW motif
Hh	hedgehog
HIF-1α	hypoxia-inducible factor-1α
HLA-DR	human leukocyte antigen—antigen D related
HSC	hematopoietic stem cells
ICAM	intercellular adhesion molecule
IDH	isocitrate dehydrogenase
IGF	insulin like growth factor
IGF1R	insulin like growth factor 1 receptor
IHH	indian hedgehog
ISCT	International Society for Cellular Therapy
JAG1	jagged 1
JMJC	jumonjiC domain-containing
MAPK	mitogen activated protein kinase
MCL-1	myeloid cell leukemia sequence 1
MDM2	mouse double minute 2
MDR1	multidrug resistance protein 1
MEK	MAPK/ERK kinase
MMP	matrix metalloproteinases
MSC	mesenchymal stem cells
MSPC	mesenchymal stem and progenitor cells
mTOR	mammalian target of rapamycin
NADPH	nicotinamide adenine dinucleotide phosphate
NF-κB	nuclear factor kappa B
NOTCH	notch homolog
OA	osteoarthritis
OPN	osteopontin
p70S6K	p70 S6 kinase
PDGFR	platelet derived growth factor receptor
PHD	prolyl hydroxylase
PI3K	phosphatidylinositol 3-kinase
PIIBNP	collagen type IIB splice variant
PD-L1	programmed cell death ligand 1
PGP-1	phagocytic glycoprotein 1
PTCH1	patched 1
r	recombinant
RANKL	receptor activator of NF-κB ligand
RANTES	regulated upon activation, normally T-expressed, and presumably secreted
RB	retinoblastoma suspectibility protein
RET	rearranged during transfection
RGS16	regulator of G-protein signaling 16
RUNX2	runt related transcription factor 2
SHH	sonic hedgehog
SMO	smoothened
SNP	single nucleotide polymorphism
SOX	SRY-related HMG box-containing
STRO-1	stromal cell surface marker-1
SZ	superficial zone
TIE2	tyrosine kinase with Ig and EGF homology domains-2
TET	ten-eleven-translocation
VEGF	vascular endothelial growth factor
VEGFR	vascular endothelial growth factor receptor
WHO	World Health Organization
WIF1	WNT inhibitory factor 1
WNT	wingless-type MMTV integration site family
